# Aging and partial body weight support affects gait variability

**DOI:** 10.1186/1743-0003-5-22

**Published:** 2008-09-19

**Authors:** Anastasia Kyvelidou, Max J Kurz, Julie L Ehlers, Nicholas Stergiou

**Affiliations:** 1 HPER Biomechanics Lab, University of Nebraska at Omaha, 6001 Dodge Street Omaha, NE 68182-0216, USA; 2 Laboratory of Integrated Physiology, University of Houston, 3855 Holman Street Houston, TX 77204-6015, USA; 3 Environmental, Agricultural and Occupational Health Sciences, College of Public Health, University of Nebraska Medical Center, 985450 Nebraska Medical Center, Omaha, NE 68198-5450, USA

## Abstract

**Background:**

Aging leads to increases in gait variability which may explain the large incidence of falls in the elderly. Body weight support training may be utilized to improve gait in the elderly and minimize falls. However, before initiating rehabilitation protocols, baseline studies are needed to identify the effect of body weight support on elderly gait variability. Our purpose was to determine the kinematic variability of the lower extremities in young and elderly healthy females at changing levels of body weight support during walking.

**Methods:**

Ten young and ten elderly females walked on a treadmill for two minutes with a body weight support (BWS) system under four different conditions: 1 g, 0.9 g, 0.8 g, and 0.7 g. Three-dimensional kinematics was captured at 60 Hz with a Peak Performance high speed video system. Magnitude and structure of variability of the sagittal plane angular kinematics of the right lower extremity was analyzed using both linear (magnitude; standard deviations and coefficient of variations) and nonlinear (structure; Lyapunov exponents) measures. A two way mixed ANOVA was used to evaluate the effect of age and BWS on variability.

**Results:**

Linear analysis showed that the elderly presented significantly more variability at the hip and knee joint than the young females. Moreover, higher levels of BWS presented increased variability at all joints as found in both the linear and nonlinear measures utilized.

**Conclusion:**

Increased levels of BWS increased lower extremity kinematic variability. If the intent of BWS training is to decrease variability in gait patterns, this did not occur based on our results. However, we did not perform a training study. Thus, it is possible that after several weeks of training and increased habituation, these initial increased variability values will decrease. This assumption needs to be addressed in future investigation with both "healthy" elderly and elderly fallers. In addition, it is possible that BWS training can have a positive transfer effect by bringing overground kinematic variability to healthy normative levels, which also needs to be explored in future studies.

## Background

Previous research has shown that gait in the elderly tends to slow down due to the natural aging of all biological systems [1-4] and/or fear of falling [5]. In addition, elderly reduce cadence, which results in an increase in the gait cycle time. They also exhibit decreased range of motion at the hip, knee, and ankle joints, as well as an increased stance time due to a longer duration of the double support [2,3]. Recently, investigators have also explored how aging affects the variability of these gait parameters. This research has revealed that the amount of variability of step length, step width, and stance time is increased in elders when compared to young subjects, or proportionally between fallers and both non-fallers and young subjects [5-10]. These studies suggested that increased amount of gait variability may be closely associated with increased risk of falling. Buzzi et al. [11] and Kurz and Stergiou [12] also examined the structure of variability present in the time series generated from gait parameters of two different age groups using nonlinear analysis. They found that the elderly exhibited significantly higher nonlinear indexes (e.g., Lyapunov exponent, Entropy). The authors suggested that gait variability degrades with physiologic aging resulting in increased randomness and noise in the neuromuscular system. They suggested that this may be one of the reasons for the increase in falling due to aging.

Body weight support (BWS) systems provide with an interactive approach to gait training, because they can be used to manipulate stability and balance by changing levels of body weight while stepping [13]. This training involves suspending a patient in a harness over the treadmill, which allows for a percentage of the patient's weight to be relieved. The training allows for repetitive locomotor training throughout a complete gait cycle. The first BWS gait training studies involved spinalized cats [14,15], in an effort to evaluate the benefits of BWS and observe whether gait could be recovered. The original results for these animal models were encouraging and thus, the usage of BWS has been expanded in human patient populations. Recent research has revealed that gait training with a BWS treadmill system has great promise to improve the ability to walk in patients with neurological diseases such as spinal cord injury [16-19], Parkinson [20] and stroke [21-28]. In Parkinson patients, BWS gait training increased speed and stride length [20]. In stroke patients, improved stepping coordination [27], bestowed greater independence [28], and reduced the fear of falling [25]. In patients with multiple sclerosis, resulted in improvements in muscle strength, spasticity, endurance, balance, walking speed, and self-reported quality of life. Possibly, these positive results could also be generated for the elderly.

In aging adults, peripheral nerves conduct impulses slower, resulting in decreased sensation, slower reflexes, and even clumsiness [29,30]. This is due to degeneration of myelin sheaths due to decreased blood flow which is accompanied with inability for axonal self-repair [29,30]. Aging is also associated with decreases in glial cells of the central nervous system [29,30]. Furthermore, aging results in significant low back pain due to degeneration of the vertebrae [29,30]. However, BWS training was credited with alleviating, or completely relieving, lower back and leg pain during ambulation [31]. Treadmill training has also been found to enhance axonal sprouting and elongation in injured peripheral nerves [32]. In addition, as the walking pattern is repeated during BWS training, the afferent input interacts with local neuromuscular control networks and modulates timing of muscle activation which helps determine the efferent pattern that is generated [33]. Since aging adults suffer from miscommunication between muscle activation and the commands given by the central nervous system, BWS gait training may help to better establish the supraspinal control of motion by increasing voluntary control of muscle groups. In fact, body weight unloading has already been used with healthy elderly through underwater treadmill walking and BWS gait resulting in improved gait speed and muscle activation patterns. [34-36] Such physiological improvements may also result in lowering to normative levels the increased gait variability that has been associated with increased risk of falling. The anticipated outcome would be fewer falls and less injury among the elderly. Clinical trials would verify such claims. However, before the employment of clinical trials, baseline studies are needed to identify the effects of BWS on elderly gait variability.

Therefore, the purpose of this study was to determine the kinematic variability of the lower extremities in young and elderly healthy females at changing levels of BWS during walking. Magnitude and structure of variability of the sagittal plane angular kinematics of the right lower extremity was analyzed using both linear (magnitude; standard deviations and coefficient of variations) and nonlinear (structure; Lyapunov exponents) measures [37]. It was hypothesized that increased levels of BWS would decrease both linear and nonlinear measures of angular kinematic variability of the hip, knee and ankle joints. It was also hypothesized that this effect would be greater for the older than the young females.

## Methods

### Subjects

Twenty young and elderly females from the community population volunteered to participate in the study. Recruiting was done by word of mouth on campus, as well as fitness facilities in the community. Ten of the subjects were between the ages of 20–35 years (mean age: 24.3, SD: 3.2 yr; mean height: 167.5, SD: 8.2 cm; mean weight: 67.3, SD: 10.6 kg), while the other ten were 70 years or older (mean age: 73.4, SD: 3.2 yr; mean height: 159.2, SD: 5.1 cm; mean weight: 72.5, SD: 10.8 kg). The subjects were in good health and capable of walking on a treadmill at a self-selected speed without holding on the handrails. None of the participants had injuries, diseases or limitations that restricted their participation in the study. Prior to participation, each subject completed an Adult Informed Consent Form approved by the university's Institutional Review Board.

### Experimental protocol

**Figure 1:**
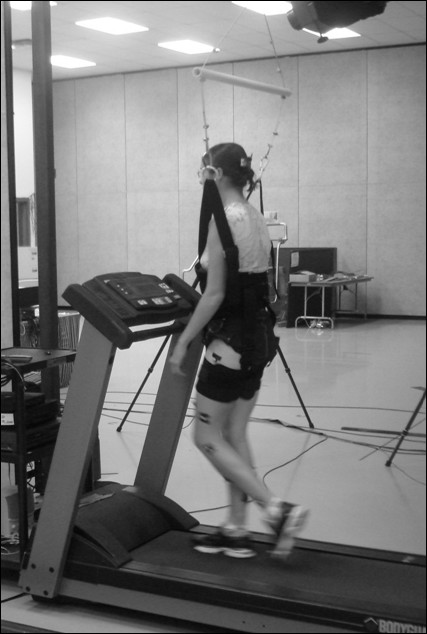
A subject walking at 1 g while being suspended by the Body Weight Support System.

A custom built BWS system was used for this investigation (Figure 1). Our suspension system and experimental methods were similar to those that have been used elsewhere to explore the influence of body weight on the mechanics of locomotion [38-45]. The BWS supplied a nearly constant upward force on the center of mass of the subject and counteracted the gravitational forces acting on the locomotive system. This was accomplished with a cable-spring-pulley system that was attached to a support vest (Biodex Medical Systems, New York, USA) and a hand winch that stretched the rubber spring elements that were in series with the cable. A spring's force is equal to the product of the spring constant (k) and the length of the spring (x). Since rubber has a nonlinear response, we adequately stretched the springs to place them within the linear region that exist at the extremes of their stress-strain elastic response. We assumed that the walking dynamics remained within this linear region due to the relatively small vertical oscillations of the subject's center of mass that occur during locomotion. A wide range of body support levels were achieved by adding additional rubber springs in parallel when the original spring would not adequately provide the proper force levels for the respective body weight support conditions. The upward lifting force values used in this experiment were measured with a piezoelectric load cell (PCB Piezotronics Inc., Depew, New York, USA) that was in series with the cable. Evaluation of the load cell during the experimental conditions indicated that our experiments were within 12% of the prescribed body weight suspensions.

Triangulations of markers were placed on the thigh, shank and foot segments. The three-dimensional positions of these markers were captured with a high-speed video capture system at 60 Hz using two Panasonic WV-CL350 video cameras. Cameras were placed approximately two meters apart and mounted at a height of 1.5 meters. One camera was placed six meters from the center of the treadmill while the other camera was angled six meters from the back end of the treadmill. The cameras were interfaced with a Peak Performance Motus 4.0 system (Peak Performance Technologies, Inc., Englewood, CO, USA). Data collection by the video tracking system was triggered by a manual transistor/transistor/logic switch. The two video views were time-synchronized by the switch that initiated data transmission. A standing calibration was also used to correct for misalignment of the markers with the local coordinate system of each of the lower extremity segments [46]. This was accomplished by having the subjects stand in a calibration fixture that was aligned with the global reference system. The calibration fixture positioned the subject's lower extremity in an anatomical position.

The subject was snuggly fitted in the body weight suspension vest and the investigator secured the leg straps that extended from the vest. In order to minimize the vest shifting up the torso with increased levels of BWS, air was hand-pumped into the right and left chambers of the vest until it was comfortably tight. Each subject performed four conditions of treadmill walking at 1 g, 0.9 g, 0.8 g, and 0.7 g (0%, 10%, 20% and 30%) BWS levels, with each condition videotaped for two minutes. The upper BWS level of 30% was selected based on recommendations from prior investigations [28,47] that indicate that the gait kinematics are least distorted at 30% BWS or below. Subjects were not allowed to hold onto the handrails during gait. The treadmill was started at the slowest available speed and five minutes were provided for the subject to become familiar with walking on the treadmill. During this time the subject established a normal gait pattern and self-selected walking speed, which became the speed used throughout the session. After this period, the treadmill was stopped to connect the BWS vest to the pulley system. The subject then resumed treadmill walking with 0% BWS (full weight bearing) at the previously selected speed. For the next minute, various levels of BWS were applied to allow the subject to experience mechanical uploading of body weight. The subjects were given sufficient time to experience different levels of BWS, since it has been shown that there is a short accommodation period when walking in reduced gravity conditions [44].

For all four BWS conditions, subjects walked at their self-selected speed determined earlier in the session. The level of BWS for the first condition was always set at 0%. Thereafter, the level of BWS was randomly adjusted to 10%, 20%, or 30% with each level being tested once. Brief breaks occurred between conditions for adjustment of BWS level. The treadmill emergency stop pull was accessible to the subject at all times.

### Data analysis

The markers were digitized using the Peak Performance Technologies Motus 4.0 system (Peak Performance Technologies, Inc., Englewood, CO, USA). The unfiltered time series of the marker position data were then exported from Peak system and was further analyzed using laboratory software developed in Matlab (Mathworks Inc., MA, USA). It has been found that filtering of the displacement data is not essential in our case because the data were not differentiated for the calculation of derivatives (velocity and acceleration; [48]).

Relative joint angles were calculated from the corrected marker positions by the methods described by Vaughn et al. [49] and Nigg et al. [46]. The minimum and maximum joint angles of the hip, knee and ankle were identified for each gait cycle for each condition. The range of motion (ROM) was calculated by subtracting the maximum and minimum values for each gait cycle. Two parameters that measure magnitude of variability, the standard deviation (SD) and coefficient of variation (CV), were estimated for each of the respective variables. In the present study joint kinematic variability was examined, because it has been shown that variability of stride characteristics offers a less sensitive measure of differences between groups than does variability of the joint kinematics [50].

A parameter that can characterize the structure of variability in a time series, the Lyapunov exponent (LyE), was also estimated from the continuous data of the gait cycles. In order to proceed with the calculation of LyE, it was required to estimate the embedded dimension or the dimension of the space that it lies in. In the present study, the estimation of the embedded dimension was performed using the global false nearest neighbor (GFNN) analysis [51]. The GFNN calculation revealed that seven embedded dimensions is required to reconstruct the state space from a given time series. The estimation of the embedded dimensions value allowed the calculation of the LyE, which is a measure of the divergence of the data trajectories in phase space, where the phase space is an n-dimensional space with n being large enough to unfold the attractor state [51]. The LyE describing purely sinusoidal data with no divergence in the data trajectories is zero because the trajectories overlap rather than diverging in phase space. This shows minimum variability over time in the data. The LyE for random noise which has a lot of divergence in the data trajectories is approximately larger than 0.5. This shows maximum variability over time in the data. Chaotic data will be described by a LyE between these two extremes [37,51]. The LyE has been previously used with gait time series data to characterize the underlying structure of variability during movement [11,52-56]. The numerical value of the largest LyE for each kinematic data time series and for each subject was calculated with the Chaos Data Analyzer (Professional Version, Physics Academic Software; [57]).

### Statistical analysis

A two way mixed (BWS level by age) analysis of variance with the BWS level as the repeated factor was performed on the subject means for SD, CV and LyE for the dependent variables of the hip, knee, and ankle ROM. A Tukey multiple comparison post hoc analysis was performed when significant differences were identified. An independent Student t-test was also used to compare the treadmill speed between the two age groups. The SPSS (Base 12.0, SPSS Inc., Chicago, IL) software package was used to perform the statistical analysis. The level of significance was set at 0.05.

## Results

As expected there was a significant difference (t = 3.4; p = 0.004; df = 18) in the self-selected walking speed between young (2.5 mph) and elderly (1.8 mph). The self-selected walking speed of the elderly was significantly slower.

### Standard deviation and coefficient of variation

**Figure 2:**
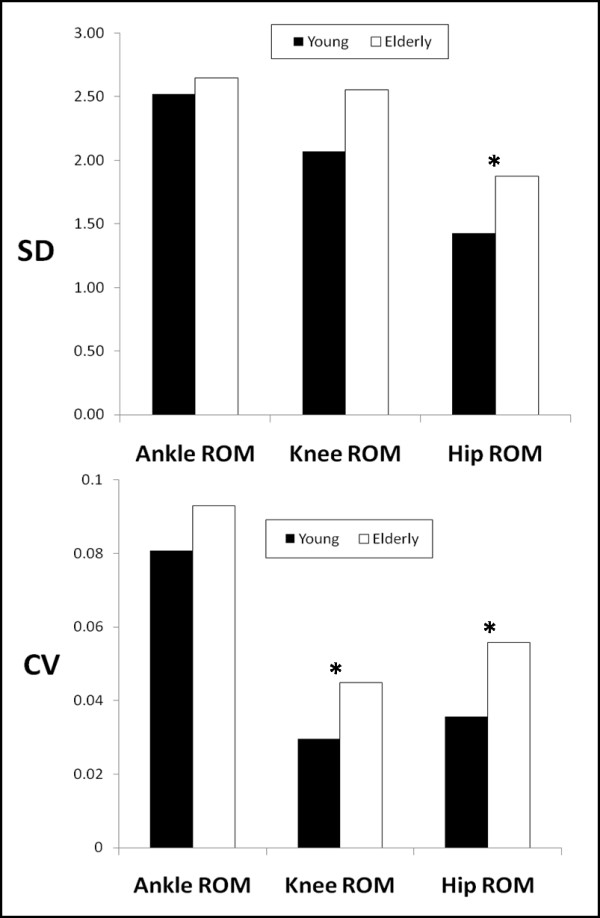
**The magnitude of hip and knee kinematic variability is increased due to age as revealed with the SD and CV analysis.** In the above graph, the statistical differences found in SD and CV values due to age are indicated with an asterisk.

No significant interactions were found between the two factors for any of the dependent variables examined (Table 1). The age factor had a significant effect for both the SD and the CV of the kinematic data. In detail, the results showed significant differences for the hip ROM SD (F(1,18) = 4.5; p = 0.048) and CV (F(1,18) = 7.8; p = 0.012), and the knee ROM CV (F(1,18) = 6.8; p = 0.017) (Figure 2). The hip and knee kinematic data indicated that the elderly had higher variability than the young in the respective joints. No significant differences were found in the variability of the ankle ROM between the young and the elderly groups.

**Table 1:** Means of SD, CV and LyE for all dependent variables

	**Young**				**Elderly**			
**BWS levels**	**0%**	**10%**	**20%**	**30%**	**0%**	**10%**	**20%**	**30%**
*Linear*								
**SD**								
Ankle ROM	2.348	2.392	2.569	2.744	2.652	2.205	2.522	3.205
Knee ROM	2.768	1.785	1.883	1.856	2.891	2.324	2.502	2.492
Hip ROM	1.360	1.296	1.510	1.531	1.985	1.712	1.818	1.994
**CV**								
Ankle ROM	0.070	0.073	0.086	0.092	0.116	0.094	0.111	0.131
Knee ROM	0.033	0.027	0.029	0.029	0.050	0.041	0.044	0.045
Hip ROM	0.031	0.031	0.040	0.040	0.052	0.049	0.054	0.068
*Nonlinear*								

**LyE**								
Ankle	0.110	0.110	0.122	0.131	0.128	0.114	0.121	0.129
Knee	0.094	0.092	0.102	0.102	0.110	0.106	0.110	0.113
Hip	0.080	0.083	0.097	0.093	0.088	0.086	0.092	0.095

Means of SD (standard deviation), CV (coefficient of variation) for the dependent variables of the hip, knee, and ankle ROM, as well as the LyE (Lyapunov Exponent) values for the ankle, knee and hip joint for both age groups and all body weight support levels.

**Figure 3:**
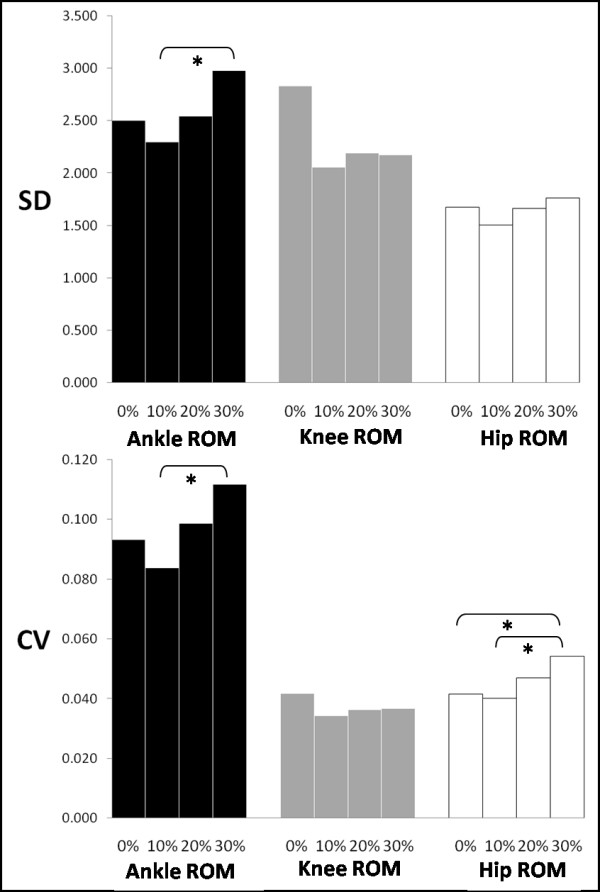
**The magnitude of variability is generally increased due to level of BWS.** The above graph, the statistical differences found in SD and CV values due to BWS level are indicated with an asterisk.

With respect to the main effect of the BWS factor, significant differences were found for the hip ROM CV (F(3,54) = 4.9; p = 0.004) and the ankle ROM SD (F(3.54) = 3.2; p = 0.031) and CV (F(3,54) = 3.9; p = 0.012) (Figure 3). In detail, hip ROM CV increased significantly from 0% to 30% BWS and 10% to 30% BWS. Ankle ROM SD and CV increased significantly from 10% to 30% BWS.

### Lyapunov exponent

**Figure 4:**
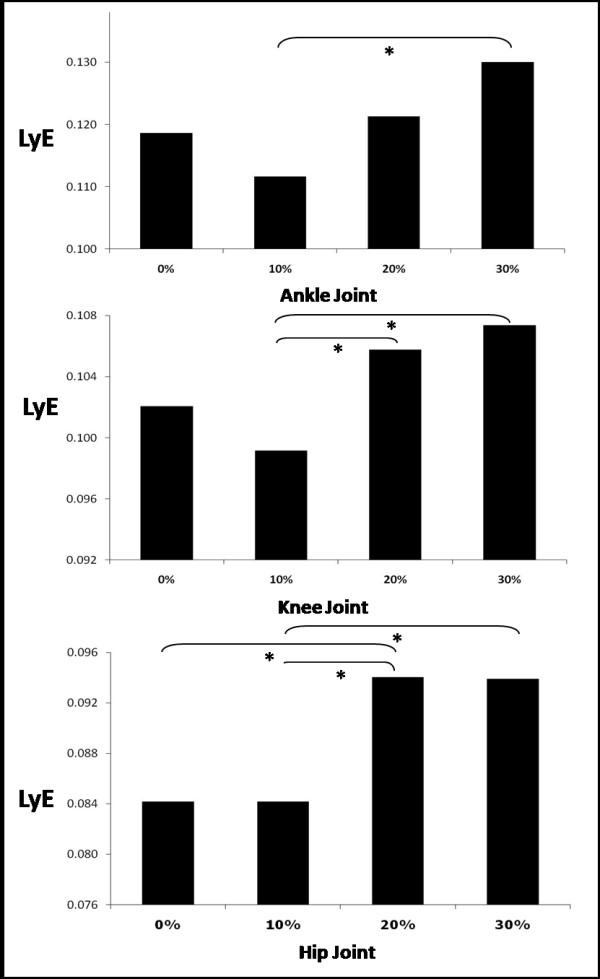
**The structure of the variability found in the time series of the kinematic parameters evaluated, changed due to level of BWS.** Higher levels of BWS revealed more divergence in the time series and more variability. The above graph includes the statistical differences found (with asterisk) in LyE values due to BWS level at each joint.

No significant interactions were found between the two factors for any of the dependent variables examined. Significant differences were identified between the BWS conditions, but not due to the age factor (Figure 4). For the ankle joint, LyE at 10% BWS was found significantly (F(3,54) = 4.9; P = 0.007) smaller than 30% BWS. At the knee joint, LyE at 10% BWS was found significantly (F(3,54) = 4.3; P = 0.012) smaller than 20% and 30% BWS respectively. Lastly at the hip joint, LyE at 20% BWS was found significantly (F(3,54) = 5.6; P = 0.004) larger than 0% and 10% BWS, and LyE at 30% was found significantly larger than 10% BWS.

## Discussion

The purpose of this study was to determine the kinematic variability of the lower extremities in young and elderly healthy females at changing levels of BWS during walking. Specifically, measures of the magnitude (linear; SD, CV) and the structure of variability (nonlinear; LyE) were employed to examine the variability present in the joint kinematic data. It was hypothesized that increased levels of BWS would decrease both linear and nonlinear measures of angular kinematic variability of the hip, knee and ankle joints. It was also hypothesized that this effect would be greater for the older than the young females. Overall our results refuted our first hypothesis showing that increased levels of BWS resulted in increased variability. Our second hypothesis was supported by the results derived from the amount of variability measures. However, our results derived from the structure of variability in the time series showed no changes due to age. Also, no significant interactions were found between the two factors for any of the dependent variables examined.

The analysis of the magnitude of variability suggested that the elderly in the present study had significantly more variability at the hip joint than the young (Figure 2). A similar result but not significant was found by Buzzi et al. [11] for the maximum of the hip angle. These results can be attributed to the decreased leg strength and flexibility exhibited by the elderly as well as loss of somatosensation [11]. Elderly individuals present reduced leg strength, hip strength and somatosensory information [58], in addition to exhibiting abnormal hip mechanics [59,60]. Kurz and Stergiou [12] suggested that there was less certainty in the aged central nervous system when selecting hip ROM during gait, possibly due to the aging neuromuscular system, which can influence gait stability. Presumably, there was even less certainty of the elderly central nervous system during gait with BWS. Findings of the present study showed significantly more variability of the knee joint in the elderly as compared to the young females (Figure 2). This is in agreement with Buzzi et al. [11] who found that the elderly had a higher CV for the knee angle during treadmill walking. Kurz and Stergiou [12] also reported that there was less certainty in the neuromuscular system of the elderly when ROM for the knee was being selected during gait. If sensory feedback is inadequate during gait then increased variability of the knee joint might occur in the elderly. In addition, even though LyE values did not differ significantly between young and elderly individuals, the elderly had higher mean LyE values overall in comparison to the young subjects (Table 1). Based on the suggestions made by Buzzi et al. [11], these results may indicate a neuromuscular effort by the elderly to maintain the deterministic properties of gait while elements of stochastic noise are introduced due to the degradation of the nervous system.

BWS significantly affected the variability of the hip joint (Figures 3 and 4). Earlier investigations [47,61] found that higher levels of BWS lifted the center of mass and as a result hip displacement decreased as there was less need for propelling the body forward during gait. Specifically, our results differ from Threlkeld et al. [47] who found minimal variability in hip motion at comparable levels of BWS, while our findings agree with those reported by Finch et al. [61] who found variability in the hip angle to be significantly more at 30% as compared to 10% BWS. This outcome was attributed by these authors to the balance component of gait being stressed at higher levels of BWS. Another explanation may be the decreased role that limb loading plays at higher BWS levels. Decreased amounts of gravity simulation have been shown to influence the muscular activity and coordination, thus alter proprioceptive information arriving from the periphery [62]. Hence, a new locomotive solution is being sought by the neuromuscular system resulting in increased variability. This may be the case with stroke patients with impaired locomotion [28], where weight unloading may lead them to seek new locomotive solutions resulting in increased neuroplasticity and eventual improvements in gait. BWS training might have similar results with the elderly, by initially increasing their gait variability through the exploration of new solutions, but the longitudinal effect would be optimally a reduction in their gait variability. However, this assumption must be tested via a longitudinal study.

The level of BWS had less impact on the knee than it did on the hip (Figure 4). Threlkeld et al. [47] also found minimal variability of the knee angle with increased BWS. Finch et al. [61] and Hesse et al. [26] reported that with increased levels of BWS more weight was supported by the harness and there was decreased activity in the vastus lateralis, which is a muscle of the knee that is sensitive to limb loading. At higher levels of BWS, a reduction of force at the knee might have resulted in less knee involvement. Additionally, Kurz and Stergiou [12] found that there was less certainty in the elderly CNS in selecting knee ROM during gait. Our findings at 0% BWS (full weight-bearing) confirm these results.

Foot function is very important during gait. Our results indicated the BWS factor had a significant effect on the ankle variability (Figures 3 and 4). In general, variability at the ankle increased with higher BWS levels, which correlates to the findings of Threlkeld et al. [47]. Earlier investigations with spinalized cats [14], stroke patients [26], and paraplegics [27] confirm our findings. It was reported that foot placement became unsteady and uncoordinated at higher levels of BWS. However, given that BWS training decreased the variability in ankle motion among those with spinal cord injuries, perhaps similar outcomes may eventually occur for the elderly. However, such a hypothesis can only be answered via a longitudinal study.

The selection of the same speed for the various gravity levels may be considered as a limitation. When walking at different gravity levels, the stability of the system is affected. For example, it is almost impossible to walk at the same speed on the moon as on the Earth. That is the reason why astronauts on the moon move much slower as gravity is reduced. Consequently, if we had allowed the subjects to self-select their speed at each BWS level, we may have had different outcomes. We plan to explore this question in a subsequent study. However, we elected to maintain the same speed between BWS conditions, in order to remove the effect of speed on the gait variability changes due to the BWS factor. However, at the same time we allowed the two groups to walk in their self-selected pace. The older women in this study had a self-selected speed that was significantly slower than the younger women, which is in agreement with other studies [2,3,59,60]. We decided to allow the two groups to walk in their self-selected pace because we did not want to force the elderly group to walk much faster than their natural gait. This could have affected their gait variability [63], especially when we consider that in the BWS conditions they would had to maintain that same speed.

Another possible limitation of the study may arise from the fact that the elder population is less familiar with use of a treadmill. The measure of the self-selected or habitual gait speed was performed on the treadmill where it may be more influenced by fear or lack of familiarity with use of treadmill which is common among older adults. Therefore, the findings may just reflect a difference between the level-ground habitual gait speed and the treadmill habitual gait speed as opposed to an age related effect. However, the treadmill has been utilized for several reasons: a) to be able to collect data from multiple studies in order to evaluate variability, b) it is really difficult to utilize BWS without a treadmill, and c) gait training in other gait related pathologies have been utilized with a treadmill since the treadmill seems to facilitate activation of spinal cord activation centers and enhance axon regeneration in peripheral nerves [32,33].

## Conclusion

Increased magnitude of variability in gait parameters has been found to be a significant predictor of falling [5]. Another investigation by Buzzi et al. [11] has showed that elderly had altered structure of variability by presenting increased randomness in their gait patterns. They speculated that this randomness is due to increased error within the neuromuscular system. This error may lead to increased likelihood of falling due to the inability of the elderly to correctly select the appropriate gait pattern. If the aging neuromuscular system is contributing to altered gait variability and subsequent instability in the elderly, then further investigation is needed to determine whether gait training can improve gait function, and perhaps reduce the incidence of falling, among the elderly.

In the present study, we found that different levels of BWS and aging affected angular kinematic variability of the hip, knee and ankle joints. Increased levels of BWS resulted in increased linear and nonlinear measures of joint kinematic variability. If the intent of BWS training is to decrease variability in gait patterns and possibly reduce the incidence of falling, certainly this cannot be supported by our results. However, a limitation of the present study is that we evaluated only "healthy" elders and not "unhealthy" elders (i.e. fallers). Furthermore, we did not perform a training study. It is possible that after several weeks of training and increased habituation, these initial increased variability values will decrease. This assumption needs to be addressed in future investigation with both "healthy" elderly and elderly fallers. In addition, it is possible that BWS training can have a positive transfer effect by bringing overground kinematic variability to healthy normative levels, which also needs to be explored in future studies.

## Competing interests

The authors declare that they have no competing interests.

## Authors' contributions

AK was involved with data analysis, statistical analysis and manuscript preparation. MJK was involved with data collection and manuscript preparation. JLE was involved in subject recruiting and data collection. NS supervised the design and coordination of the study and manuscript preparation. All authors read and approved the final manuscript.

## References

[B1] Whittle MW (2002). Gait analysis: an introduction.

[B2] Riley PO, DellaCroce U, Kerrigan DC (2001). Effect of age on lower extremity joint moment contributions to gait speed. Gait Posture.

[B3] Ostrosky KM, VanSwearingen JM, Burdett RG, Gee Z (1994). A comparison of gait characteristics in young and old subjects. Phys Ther.

[B4] Byrne JE, Stergiou N, Blanke D, Houser JJ, Kurz MJ, Hageman  PA (2002). Comparison of gait patterns between young and elderly women: an examination of coordination. Percept Mot Skills.

[B5] Maki BE (1997). Gait changes in older adults: predictors of falls or indicators of fear?. J Am Geriatric Soc.

[B6] Owings TM, Grabiner MD (2004). Variability of step kinematics in young and older adults. Gait Posture.

[B7] Hausdorff JM, Edelberg HK, Mitchell SL, Goldberger AL, Wei JY (1997). Increased gait unsteadiness in community-dwelling elderly fallers. Arch Phys Med Rehabil.

[B8] Hausdorff JM, Rios DA, Edelberg HK (2001). Gait variability and fall risk in community-living older adults: a 1-year prospective study. Arch Phys Med Rehabil.

[B9] Brach JS, Berthold R, Craik R, VanSwearingen JM, Newman AB (2001). Gait variability in community-dwelling older adults. J Am Geriatr Soc.

[B10] Brach JS, Studenski SA, Perera S, VanSwearingen JM, Newman AB (2007). Gait variability and the risk of incident mobility disability in community-dwelling older adults. J Gerontol A Biol Sci Med Sci.

[B11] Buzzi U, Stergiou N, Kurz MJ, Hageman PA, Heidel J (2003). Nonlinear dynamics indicates aging affects variability during gait. Clin Biomech.

[B12] Kurz MJ, Stergiou N (2003). The aging human neuromuscular system expresses less certainty for selecting joint kinematics during gait. Neurosci Lett.

[B13] Barbeau H, Wainberg M, Finch L (1987). Description and application of a system for locomotor rehabilitation. Med Biol Eng Comput.

[B14] Barbeau H, Rossignol S (1987). Recovery of locomotion after chronic spinalization in the adult cat. Brain Res.

[B15] Lovely RG, Gregor RJ, Roy RR, Edgerton VR (1986). Effects of training on the recovery of full-weight-bearing stepping in the adult spinal cat. Exp Neurol.

[B16] Field-Fote EC (2001). Combined use of body weight support, functional electric stimulation, and treadmill training to improve walking ability in individuals with chronic incomplete spinal cord injury. Arch Phys Med Rehabil.

[B17] Behrman AL, Harkema SJ (2000). Locomotor training after human spinal cord injury:A series of case studies. Phys Ther.

[B18] Nymark J, DeForge D, Barbeau H, Badour M, Bercovitch S, Tomas J, Goudreau L, MacDonald J (1998). Body weight support treadmill gait training in the subacute recovery phase of incomplete spinal cord injury. J Neuro Rehab.

[B19] Protas EJ, Holmes SA, Qureshy H, Johnson A, Lee D, Sherwood AM (2001). Supported treadmill ambulation training after spinal cord injury: a pilot study. Arch Phys Med Rehabil.

[B20] Miyai I, Fujimoto Y, Ueda Y, Yamamoto H, Nozaki S, Saito T, Kang J (2000). Treadmill training with body weight support: its effect on parkinson's disease. Arch Phys Med Rehabil.

[B21] Visintin M, Barbeau H, Korner-Bitensky N, Mayo NE (1998). A new approach to retrain gait in stroke patients through body weight support and treadmill stimulation. Stroke.

[B22] Kosak MC, Reding MJ (2000). Comparison of partial body weight-supported treadmill gait training versus aggressive bracing assisted walking post stroke. Neurorehabil Neural Repair.

[B23] Nilsson L, Carlsson J, Danielsson A, Fugl-Meyer A, Hellström K, Kristensen L, Sjölund B, Sunnerhagen KS, Grimby G (2001). Walking training of patients with hemiparesis at an early stage after stroke: A comparison of walking training on a treadmill with body weight support and walking training on the ground. Clin Rehabil.

[B24] Sullivan KJ, Knowlton BJ, Dobkin BH (2002). Step training with body weight support: Effect of treadmill speed and practice paradigms on poststroke locomotor recovery. Arch Phys Med Rehabil.

[B25] Hesse S, Bertelt C, Jahnke MT, Schaffrin A, Baake P, Malezic M, Mauritz KH (1995). Treadmill training with partial body weight support compared with physiotherapy in nonambulatory hemiparetic patients. Stroke.

[B26] Hesse S, Konrad M, Uhlenbrock D (1999). Treadmill walking with partial body weight support versus floor walking in hemiparetic subjects. Arch Phys Med Rehabil.

[B27] Dietz V, Colombo G, Jensen L, Baumgartner L (1995). Locomotor capacity of spinal cord in paraplegic patients. Ann Neurol.

[B28] Hassid E, Rose D, Commisarow J, Guttry M, Dobkin BH (1997). Improved gait symmetry in hemiparetic stroke patients induced during body weight-supported treadmill stepping. J Neurol Rehabil.

[B29] Kerezoudi E, Thomas PK (1999). Influence of age on regeneration in the peripheral nervous system. Gerontology.

[B30] Snijders AH, van de Warrenburg BP, Giladi N, Bloem BR (2007). Neurological gait disorders in elderly people: clinical approach and classification. Lancet Neurol.

[B31] Joffe D, Watkins M, Steiner L, Pfeifer BA (2002). Treadmill Ambulation with partial body weight support for the treatment of low back and leg pain. J Orthop Sports Phys Ther.

[B32] Sabatier MJ, Redmon N, Schwartz G, English AW (2008). Treadmill training promotes axon regeneration in injured peripheral nerves. Exp Neurol.

[B33] Harkema SJ (2008). Plasticity of interneuronal networks of the functionally isolated human spinal cord. Brain Res Rev.

[B34] Shono T, Masumoto K, Fujishima K, Hotta N, Ogaki T, Adachi T (2007). Gait patterns and muscle activity in the lower extremities of elderly women during underwater treadmill walking against water flow. J Physiol Anthropol.

[B35] Thomas EE, De Vito G, Macaluso A (2007). Speed training with body weight unloading improves walking energy cost and maximal speed in 75- to 85-year-old healthy women. J Appl Physiol.

[B36] Thomas EE, De Vito G, Macaluso A (2007). Physiological costs and temporo-spatial parameters of walking on a treadmill vary with body weight unloading and speed in both healthy young and older women. Eur J Appl Physiol.

[B37] Stergiou N, Buzzi UH, Kurz MJ, Heidel J (2004). Nonlinear Tools in Human Movement. Innovative analyses of human movement.

[B38] Teunissen LP, Grabowski A, Kram R (2007). Effects of independently altering body weight and body mass on the metabolic cost of running. J Exp Biol.

[B39] Grabowski A, Farley CT, Kram R (2005). Independent metabolic costs of supporting body weight and accelerating body mass during walking. J Appl Physiol.

[B40] Chang YH, Hamerski CM, Kram R (2001). Applied horizontal force increases impact loading in reduced-gravity running. J Biomech.

[B41] Donelan JM, Kram R (2000). Exploring dynamic similarity in human running using simulated reduced gravity. J Exp Biol.

[B42] Chang YH, Huang HW, Hamerski CM, Kram R (2000). The independent effects of gravity and inertia on running mechanics. J Exp Biol.

[B43] Griffin TM, Tolani NA, Kram R (1999). Walking in simulated reduced gravity: mechanical energy fluctuations and exchange. J Appl Physiol.

[B44] Donelan JM, Kram R (1997). The effect of reduced gravity on the kinematics of human walking: a test of the dynamic similarity hypothesis for locomotion. J Exp Biol.

[B45] Kram R, Domingo A, Ferris DP (1997). Effect of reduced gravity on the preferred walk-run transition speed. J Exp Biol.

[B46] Nigg BM, Cole GK, Nachbauer W (1993). Effects of arch height of the foot on angular motion of the lower extremities in running. J Biomech.

[B47] Threlkeld AJ, Cooper LD, Monger BP, Craven AN, Haupt HG (2003). Temporospatial and kinematic gait alterations during treadmill walking with body weight suspension. Gait Posture.

[B48] Giakas G, Baltzopoulos V (1997). Optimal digital filtering requires a different cut-off frequency strategy for the determination of the higher derivatives. J Biomech.

[B49] Vaughn CL, Davis BL, O'Connor JC (1999). Dynamics of human gait.

[B50] Barrett R, Noordegraaf MV, Morrison S (2008). Gender differences in the variability of lower extremity kinematics during treadmill locomotion. J Mot Behav.

[B51] Abarbanel HDI (1996). Analysis of observed chaotic data.

[B52] Kurz MJ, Stergiou N (2007). Do horizontal propulsive forces influence the nonlinear structure of locomotion?. J Neuroeng Rehabil.

[B53] Moraiti C, Stergiou N, Ristanis S, Georgoulis AD (2007). ACL deficiency affects stride-to-stride variability as measured using nonlinear methodology. Knee Surg Sports Traumatol Arthrosc.

[B54] Stergiou N, Moraiti C, Giakas G, Ristanis S, Georgoulis AD (2004). The effect of the walking speed on the stability of the anterior cruciate ligament deficient knee. Clin Biomech.

[B55] Buzzi UH, Ulrich BD (2004). Dynamic stability of gait cycles as a function of speed and system constraints. Motor Control.

[B56] Yoshino K, Motoshige T, Araki T, Matsuoka K (2004). Effect of prolonged free-walking fatigue on gait and physiological rhythm. J Biomech.

[B57] Sprott JC, Rowlands G (1992). Chaos data analyzer.

[B58] Low Choy NL, Brauer SG, Nitz JC (2007). Age-related changes in strength and somatosensation during midlife: rationale for targeted preventive intervention programs. Ann NY Acad Sci.

[B59] Judge JO, Davis RB, Õunpuu S (1996). Step length reductions in advanced age: the role of ankle and hip kinetics. J Gerontol A Biol Sci Med Sci.

[B60] Kerrigan DC, Todd MK, Groce UD, Lipsitz LA, Collins JJ (1998). Biomechanical gait alterations independent of speed in the healthy elderly: evidence for specific limiting impairments. Arch Phys Med Rehabil.

[B61] Finch L, Barbeau H, Arsenault B (1991). Influence of body weight support on normal human gait: development of a gait retraining strategy. Phys Ther.

[B62] Harkema SJ, Hurley SL, Patel UK, Requejo PS, Dobkin BH, Edgerton VR (1997). Human lumbosacral spinal cord interprets loading during stepping. J Neurophysiol.

[B63] Jordan K, Challis JH, Newell KM (2007). Walking speed influences on gait cycle variability. Gait Posture.

